# Integrating assisted partner notification within HIV prevention service package for people who inject drugs in Pakistan

**DOI:** 10.1002/jia2.25317

**Published:** 2019-07-19

**Authors:** Maimoona Malik, Muhammad S Jamil, Cheryl C Johnson, Muhammad S Pasha, Joumana Hermez, Salman ul H Qureshi

**Affiliations:** ^1^ Nai Zindagi Trust Islamabad Pakistan; ^2^ World Health Organization Geneva Switzerland; ^3^ World Health Organization Country Office Islamabad Pakistan; ^4^ World Health Organization Eastern‐Mediterranean Regional Office Cairo Egypt

**Keywords:** HIV testing, partner notification, community‐based, key populations, people who inject drugs

With 20,000 new HIV infections in 2017, Pakistan has the second fastest growing HIV epidemic in Asia Pacific 1. Several HIV outbreaks have occurred in the past years and the most recent outbreak was reported in April 2019 2. In the past month since the initial outbreak report, at least 186 people were newly diagnosed with HIV in one district 2. It is currently estimated that only 15.0% of people with HIV have been diagnosed and only 8.0% are receiving treatment 1.

The HIV epidemic in Pakistan is concentrated among key populations, particularly people who inject drugs (PWID) and their partners. In 2017, a population‐based survey estimated HIV prevalence among PWID was 38.4%, compared to 0.1% in the general population 1, 3. Despite this, only 39.3% of PWID tested for HIV in the past 12‐months 1, 3. The ability to reach PWID and their partners with HIV testing, prevention and treatment through traditional services is largely hindered by stigma, discrimination and policies which criminalize drug use and possession 1.

Partner notification is an effective case finding strategy for PWID and their sexual and drug injecting partners, as well as an opportunity to reduce HIV transmission to uninfected partners 4, 5. In 2016, the World Health Organization (WHO) recommended voluntary aPN services for all people with HIV as part of a comprehensive package of testing and care 5. Under an aPN model, a provider obtains consent from a person with HIV to directly contact and offer voluntary HIV testing to their sexual and/or drug injecting partner(s) 5.

High HIV burden and poor access to treatment and prevention among PWID in Pakistan has led spouses and intimate partners of PWID to be at high ongoing risk of HIV acquisition 1, 6. For the last thirty years, The Nai Zindagi Trust, a local organization founded by people affected by drug use and HIV, has been implementing a range of HIV services, including testing and aPN for PWID and their spouses, needle‐sharing and other sexual partners in Pakistan. This viewpoint describes how Nai Zindagi has been able to implement an aPN model among PWID in Pakistan since 2012.

Nai Zindagi's HIV testing services for PWID are delivered in the community by trained peer outreach workers. These services target street‐based PWID who are mostly male. All PWID with HIV are offered voluntary aPN whereby a trained female outreach worker (FORW) visits their home to offer HIV testing. These services are focused on female spouses of married PWID and are conducted only with the consent of the index (HIV positive) PWID client.

In Pakistan, joint or extended family systems, where all members of a family (parents, siblings, spouse and children of married male members) live in a household are prevalent. One in five of all married HIV positive PWID in Nai Zindagi's database report living in a joint family system. For providing aPN to the spouses of these PWID, it is critical for FORWs to engage and sensitise the mother‐in‐law of the spouse (index PWID's mother), who typically manages the domestic affairs, before approaching the spouse. Once access to the spouse of the index PWID client is obtained, FORWs register them as well as any children.

With the consent of the index PWID, FORWs disclose the index PWID's HIV positive status to the spouse and offer rapid HIV testing. Those testing negative are counselled regarding HIV transmission risks and offered prevention services to help them stay HIV negative. Those who are diagnosed with HIV (per national testing algorithm) are provided post‐test counselling and referred to a public ART centre. FORWs also provide regular home visits, ongoing social support, and tailored prevention and/or treatment adherence support including family planning and prevention of mother‐to‐child HIV transmission (PMTCT). HIV negative spouses are offered HIV testing every three months through home visits. Client confidentiality is maintained by keeping all records in secure computer files in Nai Zindagi's information management system.

Between January 2012 and December 2018, 9178 married HIV positive index PWID who had a female spouse were identified. To provide partner services to the spouses of index PWID who provided consent, FORWs undertook a total of 125,717 home visits, including follow‐up visits. FORWs successfully registered 5660 (61.7%) of these spouses. The gap in the number of married PWIDs and spouses registered is due to: difficulty in locating and accessing the spouses (14.8%); separation between partners (9.8%); being outside project coverage area (7.7%) and PWID declining partner services for their spouse (6.0%).

Of the female spouses registered, almost one‐third (29%) were aged under 30 years and three quarters (72%) had no education. At registration, 90% had at least one child, 52% reported sexual intercourse in the past month, and 86% did not use a condom at their last sexual intercourse.

FORWs performed a total of 24,598 HIV tests among registered spouses. Of the registered spouses, 4131 (72.9%) were tested at least once between 2012 and 2018 (Figure 1). A total of 365 (8.8%) were found HIV positive and of those, 287 (78.6%) were successfully linked to ART. Based on self‐report and/or pill count by FORWs, almost all HIV positive spouses on ART were adherent and taking their medications regularly (n=276, 96%).

**Figure 1:**
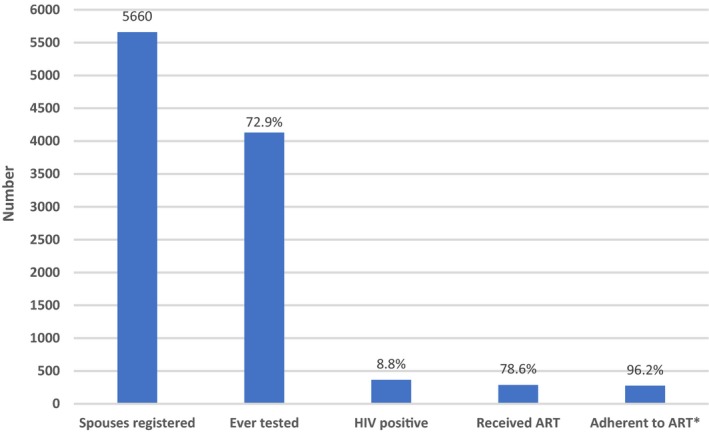
**HIV cascade among unique female spouses of index male HIV positive PWID, January 2012 to December 2018** ART, antiretroviral therapy; PWID, people who inject drugs. *Based on self‐report and/or pill count.

FORWs have encountered several challenges when conducting home visits to provide partner services and maintaining follow up. These include high levels of migration among index PWID clients, partner separation and logistical issues including lack of reliable public transportation and risks to the safety of FORWs. In some instances, when FORWs visit index PWID's home with their consent, access to the spouse is denied by family members due to sensitivities of joint family system.

Other barriers include index PWID's concern about disclosure and how their partner and family members might react to their HIV status; fear of stigma, discrimination and perusal by law enforcement agencies as drug use remains illegal in Pakistan.

While all aPN services delivered among PWID are voluntary and confidential, working within complex family systems is a challenge for Nai Zindagi staff. Additional time and efforts are needed to engage mothers‐in‐law, to obtain permission to implement partner testing. Despite having the signed consent of index PWID, without the consent of the mother‐in‐law, FORWs are often denied permission to see the spouse and are unable to deliver aPN services. In such cases, a FORW offers support to the index PWID to disclose their HIV positive status to the mother‐in‐law and other family members. The FORW then sensitizes the mother‐in‐law and family about HIV, the importance of HIV testing and seeks her permission to offer HIV testing to the spouse. Through these efforts, Nai Zindagi has successfully navigated complex family systems and reached many spouses of PWID with HIV testing services.

Given the growing HIV epidemic in Pakistan 1, opportunities to reach PWID and their sexual and drug injecting partners can no longer be missed. Despite the challenges, the Nai Zindagi programme demonstrates that even in complex settings, aPN amongst PWID and their spouses is an effective and feasible case finding strategy. More efforts are needed to replicate, scale‐up and sustain community‐led aPN models particularly among key populations and their partners.

## Competing interests

The authors have no competing interests to declare.

## Authors’ contributions

M.M. coordinated data collection, manuscript draft and submission. M.S.J. analysed and interpreted the data and contributed to manuscript draft. C.C.J. provided technical inputs and assisted with manuscript development. M.S.P. and J.H. provided technical input. S.H.Q. contributed the study design, analysis and interpretation. All authors contributed to the interpretation of results, manuscript drafts and approved the final draft of the manuscript.
